# Gene expression studies of host response to Salmonid alphavirus subtype 3 experimental infections in Atlantic salmon

**DOI:** 10.1186/1297-9716-43-78

**Published:** 2012-11-01

**Authors:** Cheng Xu, Tz-Chun Guo, Stephen Mutoloki, Øyvind Haugland, Øystein Evensen

**Affiliations:** 1 Norwegian School of Veterinary Science, Department of Basic science and Aquatic Medicine, P.O. Box 8146 Dep, Oslo 0033, Norway

## Abstract

Salmonid alphavirus subtype-3 (SAV-3) infection in Atlantic salmon is exclusively found in Norway. The salmonid alphaviruses have been well characterized at the genome level but there is limited information about the host-pathogen interaction phenomena. This study was undertaken to characterize the replication and spread of SAV-3 in internal organs of experimentally infected Atlantic salmon and the subsequent innate and adaptive immune responses. In addition, suitability of a cohabitation challenge model for this virus was also examined. Groups of fish were infected by intramuscular injection (IM), cohabited (CO) or kept uninfected in a separate tank. Samples of pancreas, kidney, spleen, heart and skeletal muscles were collected at 2, 4 and 8 weeks post infection (wpi). Pathological changes were assessed by histology concurrently with viral loads and mRNA expression of immune genes by real time RT-PCR. Pathological changes were only observed in the pancreas and heart (target organs) of both IM and CO groups, with changes appearing first in the pancreas (2 wpi) in the former. Lesions with increasing severity over time coincided with high viral loads despite significant induction of IFN-α, Mx and ISG15. IFN-γ and MHC-I were expressed in all tissues examined and their induction appeared in parallel with that of IL-10. Inflammatory genes TNF-α, IL-12 and IL-8 were only induced in the heart during pathology while T cell-related genes CD3ε, CD4, CD8, TCR-α and MHC-II were expressed in target organs at 8 wpi. These findings suggest that the onset of innate responses came too late to limit virus replication. Furthermore, SAV-3 infections in Atlantic salmon induce Th1/cytotoxic responses in common with other alphaviruses infecting higher vertebrates. Our findings demonstrate that SAV-3 can be transmitted via the water making it suitable for a cohabitation challenge model.

## Introduction

Salmonid alphaviruses (SAV) are pathogens of salmonid fish causing pancreas disease (PD) and sleeping disease (SD) in Atlantic salmon (*Salmo salar* L.) and rainbow trout (*Oncorhynchus mykiss*), respectively. They represent species of single stranded, positive polarity RNA viruses belonging to the genus *Alphavirus* in the family *Togaviridae* where they are the only ones infecting fish [1,2]. At present, they have only been isolated in Europe [3] and are responsible for great economic losses in the farmed aquaculture industry [4]. SAV are grouped into 6 subtypes (SAV-1 to SAV-6) [5], with SAV-3 being the only subtype restricted to Norway [6].

Clinical signs and histopathology associated with SAV infections are detailed elsewhere [3,7] and include degeneration of the exocrine pancreas and myopathy of heart and skeletal muscles. Mortalities can range from 1% to about 48% [3,8]. Commercial vaccines in the form of injectable preparations are available despite that the protection offered is equivocal [3]. Indeed the number of PD epizootics has remained high over the years [9].

The development of efficacious vaccines depends on a good understanding of protective immune mechanisms. For SAV infections, this has not been achieved in detail and although several studies have been undertaken to examine host responses, very few have addressed in-vivo immune responses besides Desvinges and co-workers [10] who showed that phagocytic activity of head kidney leucocytes, levels of lysozymes and complement were significantly elevated following experimental infections, indicating an active immune reaction. These authors, however, failed to detect the interferon response probably due to the poor sensitivity of the method used. Interferons are the hallmark of antiviral responses in most living organisms [11] and have been shown to be important for the host response against alphaviruses in higher vertebrates [12]. They comprise three classes of cytokines (types I to III). Amongst the three types, types I (IFN α, β, ω, ε, κ) and III (λ) are directly induced by viruses [11,13-15]. For the remaining of this article, we will not differentiate between IFN subtypes.

Following entry into the host, virus nucleic acids are sensed by host pattern recognition receptors (PRR) including Toll-like receptors (TLR 3/7/8/9) in endosomes and retinoic acid inducible gene I (RIG-I), melanoma differentiation factor-5 (MDA5) and DNA-dependent activator of IFN regulatory factors (DAI) in the cytosol [16]. Once activated, the receptors signal via MyD88/TRIF adaptors (TLR) or through the mitochondrion-associated adaptor IPS-1 (RIG-I and MDA5), all culminating in the phosphorylation and translocation of interferon regulatory factors (IRF) into the nucleus where they induce transcription of IFNα and IFNβ genes resulting in the production of IFN [17-19]. Interferons exert their effects by binding to IFN receptors (IFNAR) on target cells thereby triggering signal transduction via the Janus kinase Signal transducer activator of transcription pathway [20]. This leads to the transcription of an array of antiviral genes such as Mx, ISG-15, double stranded protein kinase R (PKR) and 2’-5’oligoadenylate synthetase (OASs) [21-23]. It is also noteworthy that a positive feed-back loop exists whereby IFNα and IFNβ act through IFNAR to up-regulate virus sensing and enhance antiviral responses [16].

Through in-vitro studies, it has been shown that IFNα induces protection against SAV-3 induced-CPE in Atlantic salmon head kidney (TO) cells [24]. This is, however, dependent on the time of exposure to interferon prior to infection. Furthermore, a positive correlation between IFNα-stimulated gene Mx expression and protection of cells against SAV-induced CPE has also been demonstrated [25,26]. The situation in-vivo, however, remains poorly understood. The in-vivo environment represents a complex milieu that differs from that of in-vitro settings. For interferons, it has been shown that these environments can yield different effects on viruses [27,28]. While a recent study has shown that IFNα and its stimulated genes are up-regulated at early time (1–5 days) in the kidneys following SAV-1 infection of Atlantic salmon [29], antiviral responses in target organs remain unknown. The purpose of the present study therefore was to examine in-vivo host responses, especially IFNα and ISG, following experimental infection of Atlantic salmon with SAV-3 in target organs. Real-time PCR was used to assess gene expression changes. Although interferon expression is known to be important at early times following infection, sampling times of 2, 4 and 8 weeks following virus injection were chosen in the present study since this is when pathological changes are known to occur. Besides, interferon responses are known to play a role in the clearance of viruses even after the onset of adaptive immune responses [27]. Cohabitants were included in order to determine the suitability of such a model for fish challenges against SAV-3. Our findings demonstrate that the virus yield and pathology progress despite the expression of interferon and related genes, in conformity with earlier reports [24].

## Materials and methods

### Virus isolation and cell culture

Chinook salmon embryonic cells (CHSE-214; ATCC CRL-1681) were used for virus propagation. The cells were maintained at 20°C with L-15 media (Invitrogen, Paisley, UK) supplemented with 5% FBS, L-glutamin and gentamycin. The virus used in the present study has previously been described [24], is fully sequenced and shown to be a typical SAV-3 subtype [GenBank: JQ799139].

### Experimental challenge

Approximately 70 Atlantic salmon (*Salmo salar* L.) pre-smolts purchased from Sørsmolts AS in Sannidal, Norway and weighing 35 ± 10 g were used. The fish were healthy and the hatchery from which they were purchased had had no previous records of PD outbreaks. The fish were transported to the Norwegian School of Veterinary Science/Veterinary Institute shared wetlab by road in oxygenated bags. After 1 week acclimatization, the fish were treated with formalin (diluted 1:4000 in water) against ectoparasites for 30 min. The fish were then kept for a further week prior to the start of the experiment.

Challenging of the fish was done by first anaesthetizing them with 0.5 mL chlorobutanol per 1L of water. Thirty fish were injected intramuscularly (IM) with 0.2 mL of the virus (2 × 10^6^ TCID_50_/mL). One group of 15 uninfected fish were fin-clipped and cohabitated with the virus-injected group to document virus replication to a level that will result in virus shedding and spread through water. The control group consisted of 15 fish that were injected with L-15 medium and were kept in a separate tank from the SAV-infected fish.

### Sample collection

At 2, 4 and 8 weeks post-infection (wpi), 10 SAV-3 injected fish, 5 cohabitants and 5 control fish were sacrificed. Parallel tissues including head kidney, spleen, heart, pancreas and muscle were collected in 10% phosphate buffered formalin for histopathology and RNAlater (Sigma, St. Louis, USA) for gene expression analysis. Tissue samples preserved in formalin were fixed for a minimum of 4 days while those kept in RNAlater were stored at −80°C until required.

### Histopathology

Paraffin-embedding, sectioning and staining with hematoxylin and eosin (H&E) were done according to standard histological procedures.

### RNA isolation and cDNA synthesis

Total RNA was isolated using the RNeasy mini Kit (Qiagen, Hilden, Germany) with on-column DNase treatment according to the manufacturer’s instructions. The concentration of RNA was determined by spectrophotometry using the Nanodrop ND1000 (Nanodrop Technologies, Wilmington, USA). For each sample, 1 μg of total RNA was subjected to cDNA synthesis using the SuperScript III reverse transcriptase system (Invitrogen, Paisley, UK) and oligo(dT)_20_ primers in a total volume of 20 μL. The synthesized cDNA was diluted 5 times by adding 80 μL distilled water and stored at −20°C until further use.

### Quantitative real-time PCR

Quantitative real-time PCR was performed using the LightCycler® 480 (Roche, Mannheim, Germany) instrument. For each gene, 2 μL of cDNA was used as template in a mixture of specific primers (10 μM) and the LightCycler 480 SYBR Green I Master mix (Roche) in a final volume of 20 μL. The mixtures were first incubated at 95°C for 10 min, followed by 40 amplification cycles (10 s at 95°C, 20 s at 60°C and 8 s at 72°C). The sequences of all primers used in this study are provided in Table 1.

**Table 1:** Primers and probe sequences used for quantitative real-time PCR

**Genes**		**Primer sequence**	**GenBank accession no.**
β-actin	Fwd	CCAGTCCTGCTCACTGAGGC	AF012125
	Rev	GGTCTCAAACATGATCTGGGTCA	
SasaIFN-α	Fwd	TGGGAGGAGATATCACAAAGC	AY216594
	Rev	TCCCAGGTGACAGATTTCAT	
Mx	Fwd	TGCAACCACAGAGGCTTTGAA	U66475
	Rev	GGCTTGGTCAGGATGCCTAAT	
ISG15	Fwd	AAGTGATGGTGCTGATTACGG	AY926456
	Rev	TTGGCTTTGAACTGGGTTACA	
IFN-γ	Fwd	CTAAAGAAGGACAACCGCAG	AY795563
	Rev	CACCGTTAGAGGGAGAAATG	
TNF-α (1&2)	Fwd	AGGTTGGCTATGGAGGCTGT	NM_001123589
	Rev	TCTGCTTCAATGTATGGTGGG	NM_001123590
IL12-β	Fwd	CTGAATGAGGTGGACTGGTATG	BT049114
	Rev	ATCGTCCTGTTCCTCCG	
IL-10	Fwd	CGCTATGGACAGCATCCT	EF165029
	Rev	AAGTGGTTGTTCTGCGTT	
IL-8	Fwd	GGCCCTCCTGACCATTACT	NM_001140710
	Rev	ATGAGTCTACCAATTCGTCTGC	
CD3-ε	Fwd	TCAGGGCTCGGAAGAAGTCT	NM_001123622
	Rev	GCCACGGCCTGCTGA	
CD4	Fwd	GAGTACACCTGCGCTGTGGAAT	NM_001124539
	Rev	GGTTGACCTCCTGACCTACAAAGG	
CD8-α	Fwd	CACTGAGAGAGACGGAAGACG	NM_001123583
	Rev	TTCAAAAACCTGCCATAAAGC	
TCR-α	Fwd	GCCTGGCTACAGATTTCAGC	BT050114
	Rev	GGCAACCTGGCTGTAGTAGC	
MHC I	Fwd	CTGCATTGAGTGGCTGAAGA	AF508864
	Rev	GGTGATCTTGTCCGTCTTTC	
MHC II	Fwd	TCTCCAGTCTGCCCTTCACC	BT049430
	Rev	GAACACAGCAGGACCCACAC	
NSAV-E2*	Fwd	CAGTGAAATTCGATAAGAAGTGCAA	EF675594
	Rev	TGGGAGTCGCTGGTAAAGGT	
E2 Probe*		FAM-5’- AGCGCTGCCCAAGCGACCG- 3’-MGB	

*From Hodneland & Endresen [30].

For the viral E2 gene, the reaction mix containing 10 μL of Probe Master, 1 μL of primer-probe mix (final concentration of each primer (0.9 μM, probe 0.25 μM), 2 μL of cDNA template and 7 μL water was incubated for 10 min at 95°C, followed by 45 amplification cycles (10 s at 95°C, 30 s at 60°C and 1 s at 72°C). To calculate the absolute quantity of the virus, recombinant pGEM-T easy (Promega, Madison, USA) plasmid containing the E2 gene of SAV-3 was used to make a standard curve in nine orders of magnitude from 10^0^ to 10^8^, thus the copy number or viral cDNA was determined. The specificity of the PCR products from each primer pair was confirmed by the melting curve analysis and subsequent agarose gel electrophoresis.

The relative expression of the following genes was examined: IFN-α, Mx, ISG-15, IFN-γ, TNF-α, IL-12, IL-10, IL-8, CD3ε, CD4, CD8, TCR-α, MHC-I, and MHC-II. To calculate the gene products, the 2-CT method was used as described elsewhere [31]. All quantifications were normalized to β-actin.

### Statistical analysis

One-way ANOVA with the Bonferroni post test was performed using GraphPad Prism version 5.00 for Windows (GraphPad Software, San Diego, CA, USA). The significant level for rejection of Ho was set at *p* < 0.05.

## Results

### Pathological changes, viral load and innate immune responses

Salmonids challenged with SAV do not develop clinical signs and mortalities are absent [3,32,33] but intramuscular or intraperitoneal injection of virus has been shown to result in an infection with high replication levels in target organs [32] with reproducible pathology. These infections are typically observed from 3 weeks post challenge and onwards, but with some differences between subtypes [34]. In order to understand the dynamics of infection we studied the interaction between viral proliferation of SAV-3 and host responses. This included, firstly, the description of pathology in primary target organs (pancreas and heart) and concomitantly, an assessment of virus replication levels in these organs; secondly, an assessment of the innate host responses following infection by examination of certain cellular markers and cytokines at transcription level before attempting to characterize the ensuing adaptive immune response. Tissues tested included primary target organs of SAV-3 and also the secondary replication site (skeletal muscle). In addition we also examined virus replication and also innate and adaptive immune genes in the head kidney and spleen. The expression of genes in the group injected intramuscularly (IM) as well as cohabitants (CO) were reported as mean fold changes relative to the control group.

### Fish intramuscularly injected with SAV-3

**Figure 1:**
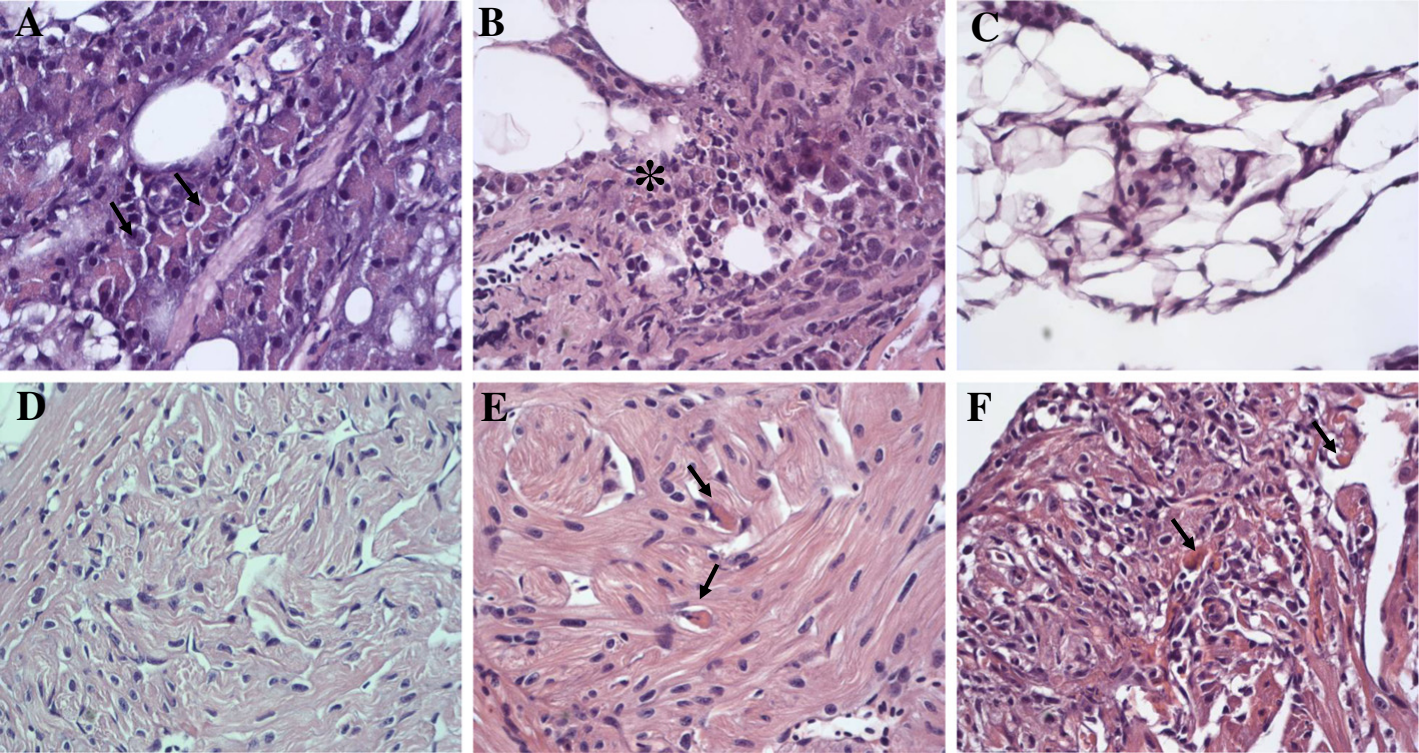
**Pathological changes in different tissues of Atlantic salmon following infection with salmonid alphavirus subtype 3.** (**A**) Pancreas, 2 wpi. Arrows = Multifocal necrosis in exocrine pancreatic cells.); (**B**) Pancreas, 4 wpi. Star = necrotic areas; (**C**) Pancreas, 8 wpi. Note depleted exocrine tissue; (**D**) Heart (ventricle), 2 wpi, normal; (**E**) Heart (ventricle), 4 wpi. Arrow = necrotic myocardial cell; (**F**) Heart (ventricle), 8 wpi, extensive cardiomyocytic necrosis and infiltration of inflammatory cells in the compact and spongious layers.

**Figure 2:**
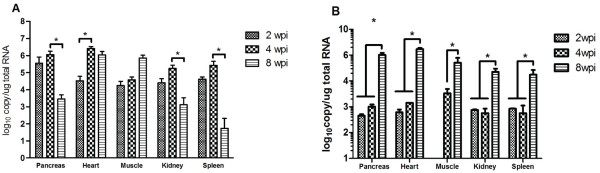
**Salmonid alphavirus subtype 3 replication in different tissues of Atlantic salmon.A**) Viral replication in different organs in intramuscularly-injected fish (average +SEM; *n* = 6 except for muscle where *n* = 3) (*p < 0.001). **B**) Viral replication in different organs of cohabitant fish at indicated time points. (average +SEM; *n* = 2 to 5) (*p < 0.001). wpi = weeks post infection. The virus was measured by the detection of copies of the E2 gene by real-time RT-PCR, expressed as log_10_ of total RNA.

**Figure 3:**
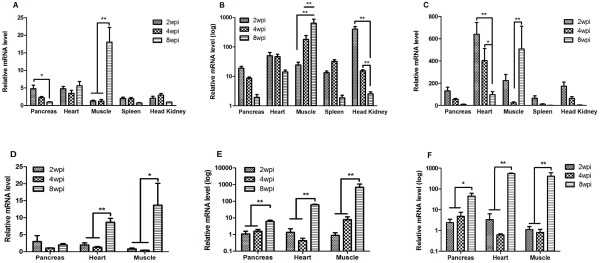
**Expression of interferon alpha and ISG in different tissues of Atlantic salmon following injection and cohabitation challenge.** IFN-α (**A**), Mx (**B**) and ISG-15 (**C**) gene expression measured at different times post challenge in injected fish. Mean values ± SEM relative to the mean of the control group (*n* = 9–10, except for muscle *n* = 3). Similar responses are presented in cohabitant fish, IFN-α (**D**), Mx (**E**) and ISG-15 (**F**) in the pancreas, heart and muscle tissues. Mean values ± SEM relative to the mean of the control group (*n* = 3–5). *p < 0.01, **p < 0.001.

In the IM group, pathological changes were first observed at 2 weeks post infection (wpi) in the pancreas characterized by acinar cell necrosis (Figure 1a), concomitant with high virus replication (Figure 2a). This was found despite high up-regulation of IFNα expression at 2 wpi (Figure 3a), increased expression of interferon-stimulated genes (ISGs) Mx (Figure 3b) and ISG-15 (Figure 3c). At 4 wpi, advanced degeneration and necrosis of acinar cells as well as inflammatory cell infiltration was observed in this group (Figure 1b), and correspondingly higher virus replication at this time point, increasing slightly from 2 to 4 wpi, albeit non-significantly (Figure 2a). Despite a higher inflammatory index in the pancreas at 4 wpi, there was no additional increase in expression of IFN-α; the expression was not different at 2 wpi compared to 4 wpi (Figure 3a). The same was observed with Mx and ISG-15 (Figure 3b and c). By 8 wpi, these pathological changes progressed to the extent that most of the exocrine pancreatic tissue had been lost (Figure 1c). This resulted in a further decline in the amount of virus (Figure 2a; *p* < 0.001), as would be expected when the tissue supporting virus replication is lost. IFN-α expression at 8 wpi (Figure 3a) had been reduced to background levels (*p* < 0.01), also in conformity with the loss/destruction of most of the exocrine pancreatic tissue at this time point. Mx and ISG-15 also fell to background levels (Figure 3b and c).

In the heart, no histopathological changes were observed at 2 wpi (Figure 1d) and the first lesions were observed at 4 wpi, seen as necrotic myocardial cells in the spongy layers (Figure 1e). IFN-α was markedly upregulated by 2 wpi but unlike the pancreas, the expression in the heart remained fairly constant (*p* = 0.05) from 2 to 4 wpi (Figure 3a). Similarly, Mx and ISG-15 was markedly upregulated by 2 wpi, 50-fold and 640-fold, respectively. Despite this, there was a sharp increase in viral load during this period (Figure 2a, *p* < 0.001). By 8 wpi, the severity of lesions in terms of necrotic cardiomyocytes increased and there was a marked infiltration of inflammatory cells not seen at 4 wpi, firstly in the ventricular spongy layer, then extending to the compact layers (Figure 1f). Occasionally, necrotic cells were also found in the atrium. The viral load remained unchanged from 4 to 8 wpi (Figure 2a). At 8 wpi there was a moderate increase of IFN-α expression (non-significant) consistent with the increase in inflammatory response at this time point (Figure 1f). The expression of Mx was lower (non-significant) and markedly down for ISG-15 (*p* < 0.001) in this organ (Figure 3b and c).

While no histopathological changes were found in the skeletal muscle over the course of the experiment, there was a steady increase in viral load from 2 to 8 wpi (Figure 2a, *p* < 0.001; 2w versus 8w and 4w versus 8 wpi). In the skeletal muscles, IFN-α was not differentially expressed at 2 and 4 wpi although both Mx and ISG-15 were significantly up-regulated at these time points (Figure 3b and c). At 8 wpi however, IFN-α expression was significantly up-regulated (*p* < 0.001), coinciding with high expression levels of Mx and ISG-15 (Figure 3b and c; *p* < 0.001).

While there was a relatively high replication of virus (1 log_10_ less than the target organs) in head kidney and spleen (Figure 2a), no lesions were observed in these organs throughout the study period.

### Cohabitant fish

In these fish, the virus was detected in all organs at all sampling times except at 2 wpi in the skeletal muscle (Figure 2b). At 2 and 4 wpi, low viral loads (about 10^2^ to 10^3^ copy numbers/μg of total RNA) were detected in tissues (Figure 2b). This translated to 2–3 log_10_ times lower than the virus detected in the IM group and these viral loads were associated with no pathology. At 8 wpi however, the virus load had increased to above 10^6^ copy numbers/μg of total RNA in the pancreas and heart, coinciding with histopathological changes. The lesions in these organs were similar to those observed in the IM group at an earlier sampling time point (Figure 1).

The expression of IFNα in all organs sampled followed a similar trend i.e. induced at 2 wpi followed by a slight drop at 4 wpi and an increase again at 8 wpi (Figure 3d). This pattern was the same as that of IFN-induced genes Mx and ISG-15 in the heart. In the pancreas and skeletal muscles however, the expression of IFN-induced genes increased with time from 2 to 8 wpi (Figure 3e and 3f). It is noteworthy that the expression of IFNα, Mx and ISG-15 increased in the presence of pathology, consistent with the observations in the IM group.

### Control fish

No mortalities were observed in any of the groups of fish in this study. No viruses were detected from the control fish, nor were any lesions observed.

### Expression of immune-related genes of adaptive immunity

The mRNA expression of IFN-γ, TNF-α, IL-12, IL-10, IL-8, CD3ε, CD4, CD8, TCR-α, MHC-I, and MHC-II were examined in the spleen, head kidney, pancreas and heart in addition to IFN-α and ISG described in the previous section.

The earliest immune response to the infection was observed in the head kidney, with IFN-γ, IL-10 and MHC-I being significantly up-regulated at 2 wpi indicating a pro-inflammatory response (Table 2). At the same time point, MHC-I was also up-regulated in the pancreas.

**Table 2:** **Relative expression of immune related genes in different tissues of Atlantic salmon after SAV**-**3 infection**

**Tissues**	**Spleen**			**Head kidney**			**Pancreas**			**Heart**		
**Genes**	**Weeks**											
	**2**	**4**	**8**	**2**	**4**	**8**	**2**	**4**	**8**	**2**	**4**	**8**
IFN-γ	0.7 ± 0.2	2.4 ± 0.5*	0.8 ± 0.1	3.0 ± 0.7*	4.1 ± 1.1*	0.8 ± 0.1	3.4 ± 0.7	7.2 ± 0.9*	1.5 ± 0.4	1.1 ± 0.2	6.3 ± 3.0*	89.3 ± 23.2*
TNF-α	0.5 **±** 0.1	0.7 **±** 0.1	1.4 **±** 0.4	1.2 **±** 0.3	1.0 **±** 0.3	0.6 ± 0.2	1.3 ± 0.3	1.4 ± 0.2	1.2 ± 0.1	2.2 ± 0.9	2.5 ± 0.3*	8.1 ± 1.5*
IL-12	0.7 ± 0.1	1.6 ± 0.2	0.8 ± 0.2	0.8 ± 0.1	1.0 ± 0.1	1.0 ± 0.1	2.2 ± 0.3	0.6 ± 0.1**	1.7 ± 0.4	1.0 ± 0.3	0.9 ± 0.2	3.3 ± 0.7*
IL-10	0.9 ± 0.1	7.5 ± 1.4*	2.8 ± 0.5*	10.0 ± 1.6*	21.2 ± 4.1*	4.7 ± 1.1*	3.1 ± 0.6	23.9 ± 6.5*	3.2 ± 0.8*	1.1 ± 0.3	4.1 ± 1.5	37.3 ± 7.6*
IL-8	1.5 ± 0.3	1.5 ± 0.6	3.0 ± 0.6*	1.0 ± 0.4	2.3 ± 0.8	1.9 ± 0.5	1.3 ± 0.2	1.7 ± 0.2	1.4 ± 0.2	1.0 ± 0.1	1.7 ± 0.3	5.7 ± 1.2*
CD3-ε	0.9 ± 0.1	0.9 ± 0.1	0.7 ± 0.1	0.6 ± 0.1	1.6 ± 0.2	1.7 ± 0.4	1.5 ± 0.3	1.4 ± 0.1	1.7 ± 0.2*	1.0 ± 0.2	1.3 ± 0.2	10.4 ± 1.9*
CD4	0.7 ± 0.1	1.3 ± 0.1	1.1 ± 0.2	0.7 ± 0.1	0.9 ± 0.1	1.1 ± 0.1	1.2 ± 0.2	1.1 ± 0.2	1.9 ± 0.3*	1.1 ± 0.5	2.5 ± 0.7	11.1 ± 1.8*
CD8	1.2 ± 0.2	1.3 ± 0.3	1.2 ± 0.3	1.0 ± 0.3	0.5 ± 0.1**	0.7 ± 0.1	**0.4** ± 0.0**	0.7 ± 0.1	2.1 ± 0.2*	1.0 ± 0.2	0.8 ± 0.3	51.6 ± 9.7*
TCR-α	1.5 ± 0.2	1.0 ± 0.1	1.0 ± 0.2	0.8 ± 0.2	0.6 ± 0.1**	0.7 ± 0.1	1.4 ± 0.1	1.4 ± 0.2	2.0 ± 0.3*	0.5 ± 0.1	0.8 ± 0.2	16.4 ± 2.5*
MHC I	1.7 ± 0.2	3.1 ± 0.2*	1.5 ± 0.2	**1.7** ± 0.2*	2.4 ± 0.2*	**1.5** ± 0.1*	**3.7** ± 0.7*	1.4 ± 0.2	2.1 ± 0.2*	1.5 ± 0.3	4.4 ± 0.5*	8.2 ± 0.8*
MHC II	1.1 ± 0.1	1.6 ± 0.2	1.2 ± 0.2	0.9 ± 0.1	1.7 ± 0.2*	1.4 ± 0.2	1.8 ± 0.3	1.4 ± 0.2	1.6 ± 0.3	0.5 ± 0.1**	1.5 ± 0.3	5.1 ± 0.8*

The results are shown as mean fold change ± standard error of virus infected group relative to the mean of the control group. Key: *Significantly up-regulated; **Significantly down regulated, otherwise not differentially induced (*P* > 0.05).

By 4 weeks, the head kidney and spleen showed a similar pattern with IFN-γ, IL-10 and MHC-I being up-regulated (Table 2). For the head kidney, MHC-II was also found to be up-regulated, although moderately while there was a down-regulation of CD8 and TCR-α (Table 2), possibly as an indication of export of these cells to the site of infection (pancreas and heart). Several pro-inflammatory genes were up-regulated in the pancreas and heart at this time point including IFN-γ (in both organs), TNF-α (heart), and MHC-I in the heart. In addition IL-10 was markedly up-regulated in the pancreas (Table 2).

At 8 wpi, fewer genes were differentially regulated in the spleen and head kidney. In the former, the expression of IL-10 continued to be induced probably to dampen the immune response while IL-8, a chemo-attractant was up-regulated at this time point (Table 2). In the head kidney, only MHC-I and IL-10 were induced at 8 wpi. In contrast, more immune-related genes were induced in the primary target organs (pancreas and heart) at 8 wpi compared to earlier time points. In the pancreas, there was a marked up-regulation of T cell markers/T cell responses (CD3, CD4, CD8, TCR, MHC-I) plus IL-10 while in the heart, all these genes as well as pro-inflammatory markers (IFN-γ, TCR-α, IL-12, IL-8 and MHC-II) were induced (Table 2).

## Discussion

In this study we show that SAV-3 infection of Atlantic salmon cause pathology in target organs alongside high viral replication despite high expression levels of IFNα mRNA and interferon-stimulated genes, ISG-15 and Mx, at early time points post challenge. Type 1 interferons are well known for the establishment of an antiviral state in neighboring uninfected cells following viral invasion in vertebrates [11,13]. This is most important during the early stages of an infection, prior to the onset of the adaptive immune response. The increase in viral loads over time in target organs (pancreas, heart and skeletal muscles) and the progression of pathology in the pancreas and heart despite the up-regulation of IFN-α, Mx and ISG-15 (Figure 3) suggest that the onset of the innate response comes too late to limit virus replication. This fits well with a previous report where treatment of cells with IFN-α at the same time as SAV-3 infection failed to protect the cells against CPE in an in-vitro model [24].

All tissues examined in the present study contained SAV-3 and the kinetics of viral loads were in general consistent with the trends of expression of IFN-α, in common with reports of previous studies done in Atlantic salmon-derived cells [24] and also with other viruses [35]. The anticipation is that SAV-3 was sensed by host cells via pattern recognition receptors such as MDA-5 and LGP2 [36] leading to the expression and induction of IFN-α and consequently ISG [28]. The trends of induction of Mx and ISG-15 by IFN-α were, on average, consistent with previous reports [24,25] while the relationship between the expression profiles was not always proportional and for ISG-15, a somewhat different expression pattern was observed in some tissues of the IM group. In one study using an in-vitro model to assess the induction of ISG by IFN-α, similar inconsistencies were observed [37]. These findings probably reflect the complexity of the interferon signaling pathways or the diversity in fish since most fish ISG are often duplicated [13], as well as the effect of IFN-α independent stimulation of ISG [38,39].

In higher vertebrates, down-stream effects of IFN-α/β induction include the increased expression of MHC I molecules [40] and activation of NK cells [41,42]. From the genes examined in the present study (Table 2), MHC I was one of the earliest genes to be induced in each organ following increased virus expression and up-regulation of IFN-α. This was consistent with previous reports where a strong association was found between IFN induction and the transcription of MHC I gene [43]. MHC I is expressed in all nucleated cells and its transcription is elevated during viral infections as a result of IFN-α/β induction and more especially, IFN-γ [44]. It is noteworthy that in the present study, MHC I was also induced in almost all tissues where IFN-γ was up-regulated (Table 2), suggesting an association between the two genes in SAV infections in Atlantic salmon as also reported by others [37].

IFN-γ is a powerful pro-inflammatory cytokine produced by cells of the lymphocyte lineage and is required for the control of intracellular pathogens [45]. Its target cells are mainly those of the monocytic origin but CD4^+^ Th1 cells are also activated [46,47]. In the present study, the expression of IFN-γ at 2 and/or 4 wpi in all organs analyzed suggests the involvement/activation of NK-like cells as part of the innate response since at these time points, there was no accompanying expression of T cell-related genes (CD4^+^, CD8^+^, TCR, CD3ε) (Table 2). This was consistent with the report that NK cells are the primary source of IFN-γ during the innate immune response [11,48]. However at 8 wpi, the expression of different genes (MHC I, CD8, TCR, MHC II, IL-12, CD4, TCR and CD3ε genes as well as the augmentation of IFN-γ) in the heart suggests a combined cytotoxic and Th1 mediated response. The pathological changes observed and the infiltration of inflammatory cells (Figure 1) fit very well with the expression of TNF-α and IL-8. It is noteworthy that the up-regulation of genes suggestive of a Th1/cytotoxic response was associated with inflammation/pathology at 8 weeks, with the reaction in the pancreas being greatly down-scaled suggesting a contraction phase. Even though IFN-γ has been shown to have a mild direct effect on SAV-3 [24], it appears to play an important role in shaping the cell mediated response or possibly contributes to the pathology seen in the target organs.

In conformity with the latter notion, it has been shown from studies of higher vertebrates that the expression of IFN-γ requires tight control since it can lead to immunopathology [49]. Furthermore, it has also been demonstrated that IFN-γ producing cells are suppressed by IL-10. IL-10 on the other hand, is itself produced by a large number of immune cells including regulatory and IFN-γ producing T cells [45,50,51]. In the present study IL-10 was consistently induced alongside IFN-γ, with the two genes showing similar trends (Table 2) that also rhymed with viral loads in individual tissues. These findings suggest the conservation of the regulation of these genes in vertebrates.

In the present study, no samples were collected prior to day 14 in the IM group, therefore, data showing the initial distribution of virus before this time is lacking. However, the viral loads of cohabitants at 2 wpi represent infection at an earlier time point compared to 2 wpi in the IM group. These results suggest that the pancreas, heart, kidney and spleen are probably all infected about the same time although the virus ultimately replicates to different levels in the different organs, with the highest load being reached in the pancreas and heart in the cohabitant group (Figure 2b). In the IM group, the virus was administered via skeletal muscle injection and it is not unlikely that an initial replication of virus occurred at the injection site, probably followed by the “draining” of the virus to other organs. The association between high viral loads and pathology in the pancreas and heart (Figures 1 and 2) suggest that the virus threshold for pathology in these organs is just above 10^6^ virus RNA copy numbers/μg of total RNA. The presence of SAV-3 in all tissues examined was consistent with previous reports that the virus has a wide range of tissue tropism in Atlantic salmon [32]. The finding of the highest viral load and pathology in the pancreas at 2 wpi in the IM group compared to other tissues is interesting especially since the viral loads culminated in all organs except the skeletal muscle at 4 weeks. This suggests that the pancreas is the most preferred site of SAV-3 replication. Several other reports allude to the pancreas as the first organ in which pathology is observed following SAV infection [3,33] and this fits with the definition of virus tropism, that being the ability of a virus to infect or cause damage to cells or tissues. On the contrary the slow and protracted increase in viral load in the skeletal muscles suggests that the organ is a site for viral persistence, in agreement with previous studies that have reported virus in this organ long after infection [32].

No lesions were observed in the skeletal muscles in the present study, in contrast with previous reports [3,7]. The viral load during the final sampling of the study was on the increase suggesting that termination at 8 weeks was probably too early, which would explain the lack of lesions. For mice infected with Sindbis virus fatalities occur when the virus invades the neurons [12]. For SAV-3 infections in Atlantic salmon, it is not clear which organs or the degree of pathology correlate with mortalities and should be a subject for further studies.

As already stated, a relationship exists between the viral load and tissue pathology, i.e. a viral load threshold has to be reached before pathology is caused. The delay in this threshold and also in the appearance of pathological changes in cohabitants in the present study compared to the IM group is consistent with a previous report where pathological changes in the former were not observed until 3 weeks following challenge [34]. These findings demonstrate that SAV-3 can spread via water, making the cohabitation challenge a possibility. The IM route of infection for SAV-3 is not natural since it is expected that fish get infected either through vectors or the water itself. Challenge studies using the cohabitation model have previously been described although they have not performed according to expectations firstly because of the difficulty to induce mortalities experimentally for SAV in general [32,34,52] and secondly because the strength of virus challenge seems to be somewhat attenuated compared to IM challenge [53]. In the present study, the presence of virus at low titers in cohabitants (Figure 2b, 2 and 4 wpi) probably allowed the fish to mount a protective immune response resulting in the delay/down regulation of pathology. Cohabitation challenge models for this virus should therefore aim to produce high quantities of infectious virus by shedders in order to enhance pathology in cohabitants or increase the number of shedders and thereby raise the infection pressure.

Finally, the rational development of vaccines offering protective immunity against pathogens relies on knowledge of basic immune responses to particular infections. This is not known in detail for SAV-3 infections in Atlantic salmon although a recent study performed by our group points to antibody responses playing a role [54]. In the present study, we demonstrate that SAV-3 infections induce mRNA transcripts of genes including IFN-α and its stimulated genes (ISG) at early time, followed by IFN-γ, TNF-α, IL-12, IL-10, IL-8, CD3ε, CD4, CD8, TCR-α, MHC-I, and MHC-II as the infection progresses. This is similar to what has been observed in other alphavirus infections in higher vertebrates [12,55], and suggests that the protection of fish against SAV-3 should be aimed at protocols that include eliciting both Th1 polarized and/or cytotoxic responses.

## Misc

Cheng Xu and Tz-Chun Guo, contributed equally to this work.

## Competing interests

The authors declare that they have no competing interests.

## Authors’ contributions

All authors designed the experimental challenge and contributed to the data analysis. XC, TG and SM took part in sampling. XC and TG did the gene expression experiments. ØE graded the histopathological changes. All authors read and approved the final manuscript.

## References

[B1] Strauss JH Strauss EG The alphaviruses: gene expression, replication, and evolution Microbio Rev 1994 58 491 562 10.1128/mr.58.3.491-562.1994 PMC372977 7968923

[B2] Weston JH Welsh MD McLoughlin MF Todd D Salmon pancreas disease virus, an alphavirus infecting farmed Atlantic salmon, Salmo salar L Virology 1999 256 188 195 10.1006/viro.1999.9654 10191183

[B3] McLoughlin MF Graham DA Alphavirus infections in salmonids - a review J Fish Dis 2007 30 511 531 10.1111/j.1365-2761.2007.00848.x 17718707

[B4] Aunsmo A Valle PS Sandberg M Midtlyng PJ Bruheim T Stochastic modelling of direct costs of pancreas disease (PD) in Norwegian farmed Atlantic salmon (Salmo salar L.) Prev Vet Med 2010 93 233 241 10.1016/j.prevetmed.2009.10.001 19931201

[B5] Fringuelli E Rowley HM Wilson JC Hunter R Rodger H Graham DA Phylogenetic analyses and molecular epidemiology of European salmonid alphaviruses (SAV) based on partial E2 and nsP3 gene nucleotide sequences J Fish Dis 2008 31 811 823 10.1111/j.1365-2761.2008.00944.x 18681902

[B6] Hodneland K Bratland A Christie KE Endresen C Nylund A New subtype of salmonid alphavirus (SAV), Togaviridae, from Atlantic salmon Salmo salar and rainbow trout Oncorhynchus mykiss in Norway Dis Aquat Organ 2005 66 113 120 16231636 10.3354/dao066113

[B7] Taksdal T Olsen AB Bjerkas I Hjortaas MJ Dannevig BH Graham DA McLoughlin MF Pancreas disease in farmed Atlantic salmon, Salmo salar L., and rainbow trout, Oncorhynchus mykiss (Walbaum), in Norway J Fish Dis 2007 30 545 558 10.1111/j.1365-2761.2007.00845.x 17718709

[B8] Crockford T Menzies FD McLoughlin MF Wheatley SB Goodall EA Aspects of the epizootiology of pancreas disease in farmed Atlantic salmon Salmo salar in Ireland Dis Aquat Organ 1999 36 113 119 10399039 10.3354/dao036113

[B9] Jansen MD Gjerset B Modahl I Bohlin J Molecular epidemiology of salmonid alphavirus (SAV) subtype 3 in Norway Virol J 2010 7 188 10.1186/1743-422X-7-188 20701761 PMC2925375

[B10] Desvignes L Quentel C Lamour F Le Ven A Pathogenesis and immune response in Atlantic salmon (Salmo salar L.) parr experimentally infected with salmon pancreas disease virus (SPDV) Fish Shellfish Immunol 2002 12 77 95 10.1006/fsim.2001.0356 11866132

[B11] Samuel CE Antiviral actions of interferons Clin Microbiol Rev 2001 14 778 809 10.1128/CMR.14.4.778-809.2001 11585785 PMC89003

[B12] Ryman KD Klimstra WB Host responses to alphavirus infection Immunol Rev 2008 225 27 45 10.1111/j.1600-065X.2008.00670.x 18837774

[B13] Verrier ER Langevin C Benmansour A Boudinot P Early antiviral response and virus-induced genes in fish Dev Comp Immunol 2011 35 1204 1214 10.1016/j.dci.2011.03.012 21414349

[B14] Zhang YB Gui JF Molecular regulation of interferon antiviral response in fish Dev Comp Immunol 2012 38 193 202 10.1016/j.dci.2012.06.003 22721905

[B15] Zou J Secombes CJ Teleost fish interferons and their role in immunity Dev Comp Immunol 2011 35 1376 1387 10.1016/j.dci.2011.07.001 21781984

[B16] Pichlmair A Sousa CRE Innate recognition of viruses Immunity 2007 27 370 383 10.1016/j.immuni.2007.08.012 17892846

[B17] Takaoka A Wang Z Choi MK Yanai H Negishi H Ban T Lu Y Miyagishi M Kodama T Honda K Ohba Y Taniguchi T DAI (DLM-1/ZBP1) is a cytosolic DNA sensor and an activator of innate immune response Nature 2007 448 501 505 10.1038/nature06013 17618271

[B18] Yoneyama M Fujita T Function of RIG-I-like receptors in antiviral innate immunity J Biol Chem 2007 282 15315 15318 10.1074/jbc.R700007200 17395582

[B19] Sun F Zhang YB Liu TK Shi J Wang B Gui JF Fish MITA serves as a mediator for distinct fish IFN gene activation dependent on IRF3 or IRF7 J Immunol 2011 187 2531 2539 10.4049/jimmunol.1100642 21795596

[B20] Schindler C Levy DE Decker T JAK-STAT signaling: from interferons to cytokines J Biol Chem 2007 282 20059 20063 10.1074/jbc.R700016200 17502367

[B21] Sadler AJ Williams BRG Interferon-inducible antiviral effectors Nat Rev Immunol 2008 8 559 568 10.1038/nri2314 18575461 PMC2522268

[B22] Zhu R Zhang YB Zhang QY Gui JF Functional domains and the antiviral effect of the double-stranded RNA-dependent protein kinase PKR from Paralichthys olivaceus J Virol 2008 82 6889 6901 10.1128/JVI.02385-07 18448522 PMC2446945

[B23] Liu TK Zhang YB Liu Y Sun F Gui JF Cooperative roles of fish protein kinase containing Z-DNA binding domains and double-stranded RNA-dependent protein kinase in interferon-mediated antiviral response J Virol 2011 85 12769 12780 10.1128/JVI.05849-11 21937641 PMC3209354

[B24] Xu C Guo TC Mutoloki S Haugland O Marjara IS Evensen O alpha interferon and not gamma interferon inhibits salmonid alphavirus subtype 3 replication in vitro J Virol 2010 84 8903 8912 10.1128/JVI.00851-10 20573808 PMC2919011

[B25] Gahlawat SK Ellis AE Collet B Expression of interferon and interferon - induced genes in Atlantic salmon Salmo salar cell lines SHK-1 and TO following infection with Salmon AlphaVirus SAV Fish Shellfish Immunol 2009 26 672 675 10.1016/j.fsi.2009.02.021 19264132

[B26] Lester K Hall M Urquhart K Gahlawat S Collet B Development of an in vitro system to measure the sensitivity to the antiviral Mx protein of fish viruses J Virol Methods 2012 182 1 8 10.1016/j.jviromet.2012.01.014 22405879

[B27] Pang KR Wu JJ Huang DB Tyring SK Baron S Biological and clinical basis for molecular studies of interferons Methods Mol Med 2005 116 1 23 16007741 10.1385/1-59259-939-7:001 PMC7121562

[B28] Zhang YG Burke CW Ryman KD Klimstra WB Identification and characterization of interferon-induced proteins that inhibit alphavirus replication J Virol 2007 81 11246 11255 10.1128/JVI.01282-07 17686841 PMC2045553

[B29] Herath TK Bron JE Thompson KD Taggart JB Adams A Ireland JH Richards RH Transcriptomic analysis of the host response to early stage salmonid alphavirus (SAV-1) infection in Atlantic salmon Salmo salar L Fish Shellfish Immunol 2012 32 796 807 10.1016/j.fsi.2012.02.001 22365992

[B30] Hodneland K Endresen C Sensitive and specific detection of Salmonid alphavirus using real-time PCR (TaqMan (R)) J Virol Methods 2006 131 184 192 10.1016/j.jviromet.2005.08.012 16202457

[B31] Livak KJ Schmittgen TD Analysis of relative gene expression data using real-time quantitative PCR and the 2(T)(−Delta Delta C) method Methods 2001 25 402 408 10.1006/meth.2001.1262 11846609

[B32] Andersen L Bratland A Hodneland K Nylund A Tissue tropism of salmonid alphaviruses (subtypes SAV1 and SAV3) in experimentally challenged Atlantic salmon (Salmo salar L.) Arch Virol 2007 152 1871 1883 10.1007/s00705-007-1006-1 17578649

[B33] McLoughlin MF Nelson RN McCormick JI Rowley HM Bryson DB Clinical and histopathological features of naturally occurring pancreas disease in farmed Atlantic salmon, Salmo salar L J Fish Dis 2002 25 33 43 10.1046/j.1365-2761.2002.00334.x

[B34] Graham DA Frost P McLaughlin K Rowley HM Gabestad I Gordon A McLoughlin MF A comparative study of marine salmonid alphavirus subtypes 1–6 using an experimental cohabitation challenge model J Fish Dis 2011 34 273 286 10.1111/j.1365-2761.2010.01234.x 21294751

[B35] Jorgensen JB Johansen A Hegseth MN Zou J Robertsen B Collet B Secombes CJ A recombinant CHSE-214 cell line expressing an Mx1 promoter-reporter system responds to both interferon type I and type II from salmonids and represents a versatile tool to study the IFN-system in teleost fish Fish Shellfish Immunol 2007 23 1294 1303 10.1016/j.fsi.2007.07.008 17804253

[B36] Chang MX Collet B Nie P Lester K Campbell S Secombes CJ Zou J Expression and functional characterization of the RIG-I-like receptors MDA5 and LGP2 in Rainbow trout (Oncorhynchus mykiss) J Virol 2011 85 8403 8412 10.1128/JVI.00445-10 21680521 PMC3147945

[B37] Sun BJ Skjaeveland I Svingerud T Zou J Jorgensen J Robertsen B Antiviral activity of salmonid gamma interferon against infectious pancreatic necrosis virus and salmonid alphavirus and its dependency on type I interferon J Virol 2011 85 9188 9198 10.1128/JVI.00319-11 21697489 PMC3165780

[B38] Collins SE Noyce RS Mossman KL Innate cellular response to virus particle entry requires IRF3 but not virus replication J Virol 2004 78 1706 1717 10.1128/JVI.78.4.1706-1717.2004 14747536 PMC369475

[B39] Noyce RS Collins SE Mossman KL Identification of a novel pathway essential for the immediate-early, interferon-independent antiviral response to enveloped Virions J Virol 2006 80 226 235 10.1128/JVI.80.1.226-235.2006 16352547 PMC1317555

[B40] Samuel CE Antiviral actions of interferon. Interferon-regulated cellular proteins and their surprisingly selective antiviral activities Virology 1991 183 1 11 10.1016/0042-6822(91)90112-O 1711253

[B41] Biron CA Nguyen KB Pien GC Cousens LP Salazar-Mather TP Natural killer cells in antiviral defense: Function and regulation by innate cytokines Annu Rev Immunol 1999 17 189 220 10.1146/annurev.immunol.17.1.189 10358757

[B42] Reiter Z Interferon - a major regulator of natural-killer cell-mediated cytotoxicity J Interferon Res 1993 13 247 257 10.1089/jir.1993.13.247 7693829

[B43] Landis ED Purcell MK Thorgaard GH Wheeler PA Hansen JD Transcriptional profiling of MHC class I genes in rainbow trout infected with infectious hematopoietic necrosis virus Mol Immunol 2008 45 1646 1657 10.1016/j.molimm.2007.10.003 18187194

[B44] Boehm U Klamp T Groot M Howard JC Cellular responses to interferon-gamma Annu Rev Immunol 1997 15 749 795 10.1146/annurev.immunol.15.1.749 9143706

[B45] Chen JZ Liu XS The role of interferon gamma in regulation of CD4(+) T-cells and its clinical implications Cell Immunol 2009 254 85 90 10.1016/j.cellimm.2008.09.001 18848698

[B46] Schmitt E Hoehn P Huels C Goedert S Palm N Rude E Germann T T helper type 1 development of naive CD4+ T cells requires the coordinate action of interleukin-12 and interferon-gamma and is inhibited by transforming growth factor-beta Eur J Immunol 1994 24 793 798 10.1002/eji.1830240403 7908633

[B47] Wakil AE Wang ZE Ryan JC Fowell DJ Locksley RM Interferon gamma derived from CD4(+) T cells is sufficient to mediate T helper cell type 1 development J Exp Med 1998 188 1651 1656 10.1084/jem.188.9.1651 9802977 PMC2212510

[B48] Shtrichman R Samuel CE The role of gamma interferon in antimicrobial immunity Curr Opin Microbiol 2001 4 251 259 10.1016/S1369-5274(00)00199-5 11378475

[B49] Cope A Le Friec G Cardone J Kemper C The Th1 life cycle: molecular control of IFN-gamma to IL-10 switching Trends Immunol 2011 32 278 286 10.1016/j.it.2011.03.010 21531623

[B50] O’Garra A Vieira PL Vieira P Goldfeld AE IL-10-producing and naturally occurring CD4(+) Tregs: limiting collateral damage J Clin Invest 2004 114 1372 1378 15545984 10.1172/JCI23215 PMC525746

[B51] O’Garra A Vieira P T(H)1 cells control themselves by producing interleukin-10 Nat Rev Immunol 2007 7 425 428 10.1038/nri2097 17525751

[B52] Andersen L Hodneland K Nylund A No influence of oxygen levels on pathogenesis and virus shedding in Salmonid alphavirus (SAV)-challenged Atlantic salmon (Salmo salar L.) Virol J 2010 7 198 10.1186/1743-422X-7-198 20727205 PMC2936311

[B53] Karlsen M Tingbo T Solbakk IT Evensen O Furevik A Aas-Eng A Efficacy and safety of an inactivated vaccine against Salmonid alphavirus (family Togaviridae) Vaccine 2012 30 5688 5694 10.1016/j.vaccine.2012.05.069 22691434

[B54] Xu C Mutoloki S Evensen O Superior protection conferred by inactivated whole virus vaccine over subunit and DNA vaccines against salmonid alphavirus infection in Atlantic salmon (Salmo salar L.) Vaccine 2012 30 3918 3928 10.1016/j.vaccine.2012.03.081 22504037

[B55] Klimstra WB Ryman KD Bernhard KA Nguyen KB Biron CA Johnston RE Infection of neonatal mice with sindbis virus results in a systemic inflammatory response syndrome J Virol 1999 73 10387 10398 10559357 10.1128/jvi.73.12.10387-10398.1999 PMC113094

